# Presenting or Spinning Facts? Deconstructing the U.S. Centers for Disease Control Statement on the Importance of Reopening Schools Under COVID-19

**DOI:** 10.3389/fpubh.2021.645229

**Published:** 2021-03-09

**Authors:** Habib Benzian, Marilyn Johnston, Nicole Stauf, Richard Niederman

**Affiliations:** ^1^Department of Epidemiology & Health Promotion, World Health Organization Collaborating Center, College of Dentistry, New York University, New York, NY, United States; ^2^The Health Bureau Ltd Consultants for Global Health, Buckingham, United Kingdom; ^3^Department of Epidemiology & Health Promotion, College of Dentistry, New York University, New York, NY, United States; ^4^Independent Researcher, Berlin, Germany

**Keywords:** COVID-19, disaster communication, public health guidance, school safety, reopening, political bias, pandemic mitigation

## Abstract

Credible, reliable and consistent information to the public, as well as health professionals and decision makers, is crucial to help navigate uncertainty and risk in times of crisis and concern. Traditionally, information and health communications issued by respected and established government agencies have been regarded as factual, unbiased and credible. The U.S. Centers for Disease Control and Prevention (CDC) is such an agency that addresses all aspects of health and public health on behalf of the U.S Government for the benefit of its citizens. In July 2020, the CDC issued guidelines on reopening schools which resulted in open criticism by the U.S. President and others, prompting a review and publication of revised guidelines together with a special “Statement on the Importance of Reopening Schools under COVID-19.” We hypothesize that this statement introduced bias with the intention to shift the public perception and media narrative in favor of reopening of schools. Using a mixed methods approach, including an online text analysis tool, we demonstrate that document title and structure, word frequencies, word choice, and website presentation did not provide a balanced account of the complexity and uncertainty surrounding school reopening during the COVID-19 pandemic. Despite available scientific guidance and practical evidence-based advice on how to manage infection risks when reopening schools, the CDC Statement was intentionally overriding possible parent and public health concerns. The CDC Statement provides an example of how political influence is exercised over the presentation of science in the context of a major pandemic. It was withdrawn by the CDC in November 2020.

## Credible Health Communication in the Time of COVID-19

Credible, reliable and consistent information to the public, as well as health professionals and decision makers, is crucial to help navigate uncertainty and risk in times of crisis and concern. The COVID-19 pandemic has generated an information surge, based on an unprecedented amount of rapidly evolving and accessible data, amplified by modern mass communication channels, oftentimes unvetted in terms of quality, truthfulness or scientific evidence. The individual's ability to distinguish real from fake or important from hyped is tested to the maximum in what the World Health Organization calls an “infodemic” (1). Decisions on whom to trust are all too often made on the basis of who shouts the loudest or gets the most social media attention, rather than on the basis of rational assessment, transparency and credibility of the source (2, 3).

Traditionally, information and health communication items issued by respected and established government agencies have been regarded as factual, unbiased and credible. The U.S. Centers for Disease Control and Prevention (CDC) is an agency that addresses all aspects of health and public health on behalf of the U.S. Government for the benefit of its citizens. The CDC's pledge is to “base all public health decisions on the highest quality scientific data that is derived openly and objectively,” which sets a high bar of scientific integrity for information and guidance that is provided for health professionals, political decision makers, media and the public at large (4).

**Figure 1:**
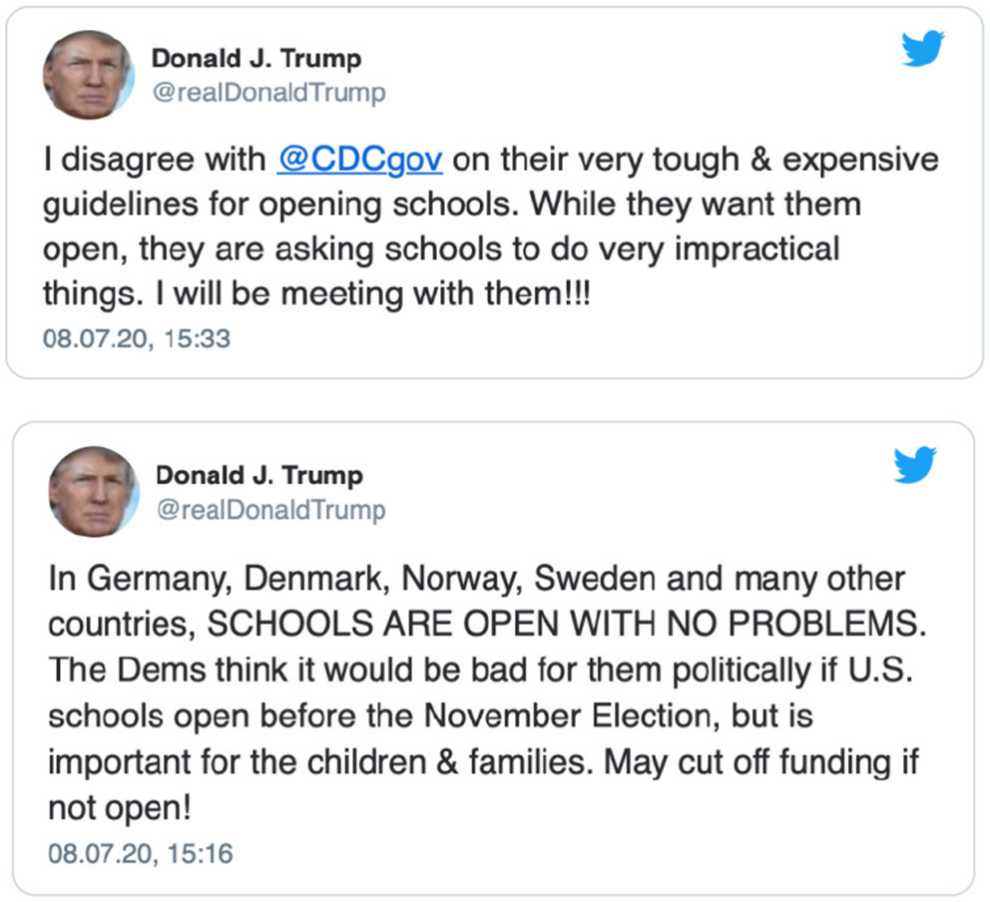
Tweets of the U.S. President related to the CDC guidelines on reopening schools (08.07.2020).

The COVID-19 pandemic has exacerbated pre-existing tensions between science and politics in the U.S. (and elsewhere), challenging the role and credibility of science and the translation of scientific advice into effective public health policy and action. Such conflicts have recently resulted in a situation where the CDC was requested to reconsider its draft guidance on reopening of schools during the COVID-19 pandemic (5). According to media reports, as well as President Donald Trump's own tweets from July 8, 2020 (Figure 1), the White House felt the original draft guidelines placed too much emphasis on the infection risks related to school reopening and threatened federal defunding of public schools unless the guidelines were reworked.

Reacting to the criticism, Vice President Mike Pence and CDC Director Robert Redfield announced that revised guidelines would be released the following week. According to the New York Times, the Department of Health and Human Services took control of the revisions with minimal input from the CDC (6). When the CDC eventually released its revised guidelines on July 23, 2020, it introduced them with a separate statement entitled “The Importance of Reopening America's Schools this Fall” (we use the term “Statement” in this paper) (7).

## Hypothesis—the CDC Statement Introduces Bias

It is our hypothesis that the introductory Statement of the revised guidance introduced politically motivated bias into a scientific discussion. To test this hypothesis we analyzed the CDC Statement, using language and contextual analysis tools to determine whether the information is presented objectively, in accordance with CDC's pledge. In this paper we do not enter into details of the politics of the relationship between the CDC and the U.S. Government. Neither do we assess the scientific evidence of the arguments put forward against or in favor of a return to regular school attendance.

## Analysis Methodology

We used a mixed-methods approach to analyze the text and online presentation context of the CDC Statement. With support of an open-source free online text analysis tool (8) (http://www.voyant-tools.org, University of Alberta, Canada, and McGill University, Canada), we undertook simple automated analyses of the Statement's main body, excluding footnote text. We analyzed word frequencies to compile a ranking of words according to frequencies; a predetermined set of stopwords was excluded, based on a standard list of English stopwords available from Voyant-tool.org). We tabulated the first five most common terms and three additional selected words related to the pandemic (“Covid-19,” “risk/risks,” “safety”). In addition, the authors manually analyzed the document structure and performed a word-by-word content, contextual and emotional connotation analysis (9). This analysis was first conducted independently by each co-author, then in a second step jointly reviewed to achieve consensus. Lastly, we analyzed the presentation of the Statement in the context of the overall COVID-19 guidance for schools on the CDC's website, specifically looking at the placement of elements, integration in the website style and format and the relation to website navigation elements. The analysis was undertaken in September 2020.

## Findings

### Choice of Document Title

The title of the CDC Statement (“The Importance of Reopening America's Schools this Fall”) is declarative, identifying the document's view, rather than an evaluation of risks and benefits.

### Document Organization and Selectivity of Topics

The Statement is structured into eight broad sections, starting with an introduction. Only the second section with the heading “COVID-19 and Children” briefly discusses the health risks of COVID-19 and the particular situation of children. From the total word count of 2,315 words only 307 are dedicated to this topic (see Table 1). The other sections expound on the value of school attendance for education, social and emotional development, safety, nutrition and physical activity. The section on safety does not deal with safety from infection risks, but with the role of the school in the context of safety from abuse and violence. Text related to serious disease complications is shifted to a footnote rather than explaining the complications in the main body of text.

**Table 1:** Document sections and respective word count/ranking and frequency of selected words in the CDC statement (excluding footnotes).

**Section**		**Word count**
Introduction		238
Covid-19 and Children		307
Educational Instruction		455
Social and emotional development		640
Safety		186
Nutrition		118
Physical Activity		166
Conclusion		178
Total word count (including subheadings)		2,315
**Rank**	**Word/s**	**Frequency**
1	School/schools	74
2	Children	54
3	Students	18
4	Learning	17
5	Covid	16
…		
116/117	Risk/Risks	3/3
…		
195	Safety	2

### Word Count and Frequency

Table 1 also shows selected words and their respective frequency of use in the Statement. The terms “school/schools,” “children,” “students,” and “learning” occupied the ranks one to four. The term “COVID” ranked 5th most frequent. The words “risk/risks” ranked 116/117th with three mentions each and the word “safety” ranked 195th with only two mentions in the entire document.

### Word Choice

The choice of words and phrasing of the Statement demonstrate vagueness and bias, as illustrated in this annotated version (annotations in square brackets) of the second paragraph of the section with the heading, “COVID-19 and Children:”

Scientific studies suggest [indicates that studies are not yet certain; no references] that COVID-19 transmission among children in schools may [indicates uncertainty] be low. International studies [no references] that have assessed how readily COVID-19 spreads in schools also reveal [the other studies do not reveal but suggest, however here it is implied that the information provided is certain] low rates of transmission when community transmission is low. [This is an important qualifier, but its relevance to decision making about reopening is not discussed.] Based on current data, [references missing] the rate of infection among younger [the age cohort is not mentioned, which would be important for making this statement more relevant] school children, and from students to teachers, has been low, especially if proper precautions are followed. [This is an important “if,” but its importance is not discussed and the details of the precautions are not provided.] There have also been few reports [vague, references missing] of children being the primary source of COVID-19 transmission among family members. [Were these children attending school?] This is consistent with data from both virus and antibody testing, suggesting [“suggesting” is not proving.] that children are not the primary drivers of COVID-19 spread in schools or in the community. No studies are conclusive, but the available evidence provides reason to believe [vague] that in-person schooling is in the best interest of students, particularly in the context of appropriate mitigation measures similar to those implemented at essential workplaces. [The appropriate mitigation measures are not discussed in this statement. This blanket assertion is not helpful to those seeking advice on how to safely reopen schools.]

### Website Presentation

**Figure 2:**
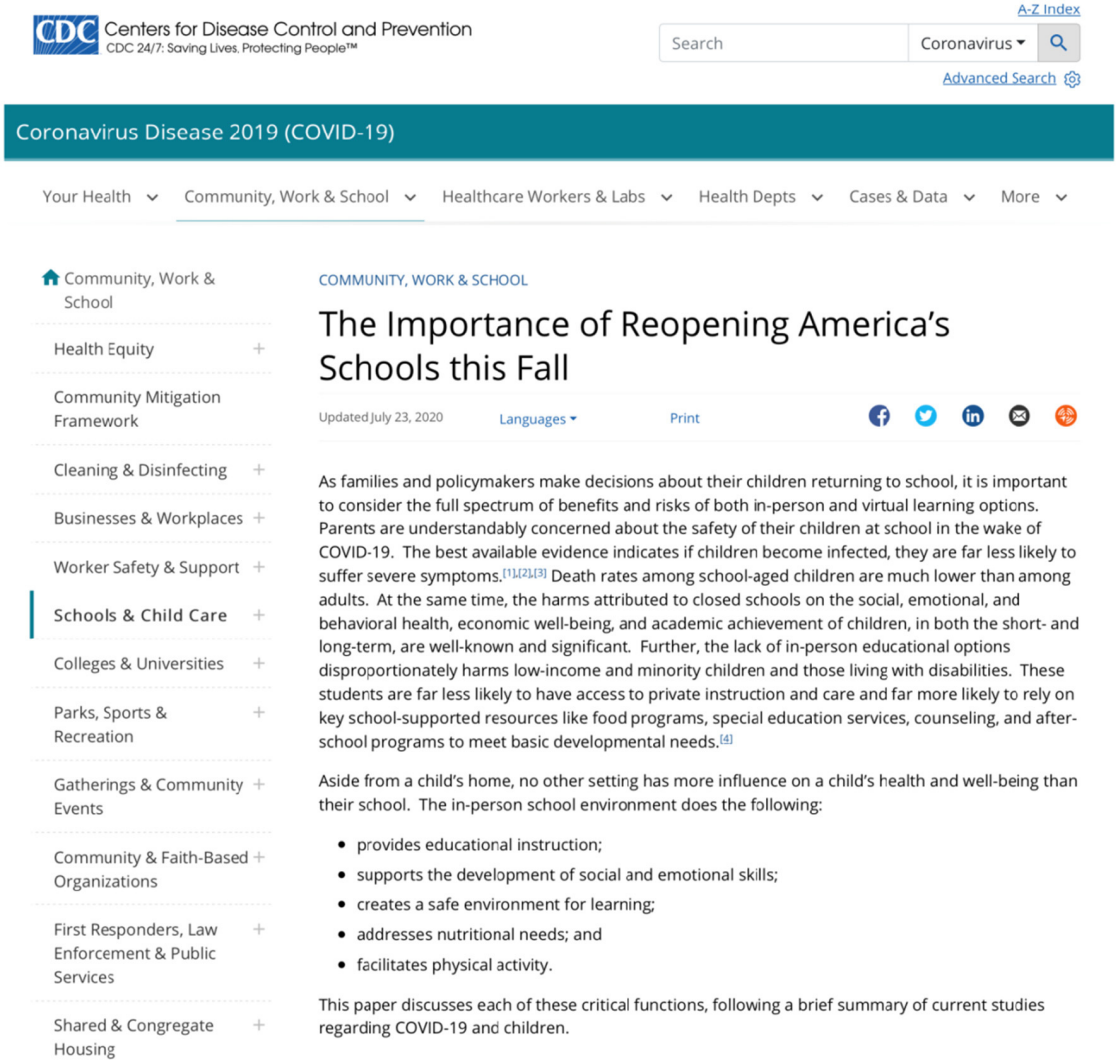
Screenshot of the CDC website featuring the Statement on *The Importance of Reopening America's Schools this Fall*.

The Statement is presented on a webpage separate from the technical guidance documents. The latter are accessible via a left-hand menu of searchable tabs, requiring additional clicks to unfold sub-menus in order to find and select guidance related to school reopening. Users reaching the website through a search engine may have difficulty finding the technical content or mistake the statement as the main guidance (see the website screenshot in Figure 2 or https://www.cdc.gov/coronavirus/2019-ncov/community/schools-childcare/reopening-schools.html).

## Discussion

The CDC Statement provides an example of how political influence is exercised over the presentation of science in the context of a major pandemic. The fact that the original draft of the CDC's guidelines on school reopening was publicly questioned by the U.S. President; that an additional statement was crafted to precede and pre-empt the revised guidelines without actually incorporating them; and that the title of the Statement itself contains no reference to “safety,” all combined suggest that there was a deliberate intent to spin the CDC's scientific guidance toward reopening of schools. Our analysis confirms this initial hypothesis and provides evidence of biased and selective presentation of science, intended to downplay the COVID-19 infection risks for children, teachers and staff returning to school. However, we acknowledge a potential and virtually unavoidable analysis bias. The extent of White House interference in CDC matters was the subject of multiple media reports and became the origin of our interest in undertaking the presented analysis (10).

Unlike an earlier CDC communication on school reopening from May 19, 2020, the new CDC Statement neither addresses the risks of reopening in detail nor discusses how to manage them safely. Instead, it elaborates at great length on the benefits of reopening and the missed benefits of not doing so. In seven of the eight sections the Statement speaks about the educational needs of children, the benefits of attending school and, in particular, the detriments of not attending, none of which is questioned by anyone. It is interesting to note that the Statement boasts about school-related benefits that have been under pressure, limited, or otherwise challenged by the Trump administration's policies aimed at weakening the public school system (11, 12).

Building on extensive evidence, several practical health communication tools have been developed for the context of pandemics and the COVID-19 pandemic in particular (13–15). Among the recognized principles of effective health communication in times of crisis is the need to transparently acknowledge concerns of the reader, as well as existing uncertainties and evolving scientific evidence (16, 17). However, the challenges and difficulties of balancing competing interests and the hard choices facing decision-makers - in short, how and when do you open schools safely during a pandemic - are left unsaid in the CDC Statement. Nowhere in the text can the reader find recommendations on when to reopen or how to reopen safely. Readers are left on their own to discover those recommendations, as they are not immediately visible and are accessible only via an interactive list of topics on the left-hand side of the CDC's web page. Navigating such a page arrangement may be familiar to someone who reads scientific publications regularly or professionally, but it may not be familiar to the parent who googles “CDC school reopening guidelines.”

Even though the CDC's technical guidelines and tool tabs on the website provide a more balanced risk-benefit analysis of reopening and offer recommendations on how to keep children, teachers and staff safe at school, they remained hidden by separating them from the Statement in an obvious effort to strengthen the case for re-opening (see Figure 2). It would have been appropriate to provide a more nuanced and less biased text by simply incorporating the word “safely reopen schools” in the title, by adding more detail on the disease risk and by highlighting existing evidence on school-based mitigation measures. As this was not done, it may imply that the CDC Statement was politically motivated and biased to present information favoring reopening of schools. However, by overly emphasizing this message and practically ignoring parent, teacher, staff and family concerns about COVID-19 transmission, the Statement's authors do not provide a balanced perspective but a rather one-sided view.

Critical in the broader frame of transparency and accountability is the fact that the Statement was published under the CDC's name and brand, as part of a set of technical guidelines. The fact that the Statement was conceptualized and largely drafted by people not within the CDC, and that CDC was given limited opportunity to provide input is not communicated openly (6). Readers unfamiliar with this important political context will read and understand the Statement as part of the CDC's scientific products. Worse, this lack of context and transparency may make the reader more likely to read and understand the detailed technical guidance through the lens of prioritizing reopening, rather than through the lens of minimizing risks of infection for children, teachers, staff and families.

The Statement was withdrawn by the CDC on November 17, 2020, 2 months after our analysis presented here (18). The deletion resulted from a critical inquiry of the House of Representatives Select Subcommittee on the Coronavirus Crisis initiated in September 2020. The complete erasure of the Statement from the CDC's website without replacement (it is not even available through the site's search function) is the ultimate confirmation that the Statement was not in line with the CDC's usual standards of quality and scientific rigor.

## Conclusion

The U.S. and many other countries witnessed heated and politicized public discussions about the risks and benefits of open or closed schools amidst an ongoing pandemic with continued high rates of disease transmission. The importance of reopening school is widely recognized and acknowledged, but it is only safe if available scientific guidance and practical evidence-based advice on how to manage infection risks when reopening schools are duly observed. The additional Statement simply overrides all public health concerns by pushing technical details and balanced risk assessments into the background. The fact that the Trump administration felt the need to add an interpretative layer to the CDC's science-based guidance is an expression of its general disregard for science and its preference that favors economic returns over potential harm to children, teachers, school staff and families (19). Political interference by the White House undermines the CDC's credibility as a leading public health agency, making it difficult for the agency to be viewed as a credible provider of equipoised guidance (20).

In the context of effective health communication in a pandemic, “wise politicians realize the limits to their knowledge and their ability to spin things in the real world. This is a disease, it doesn't care what we think and say, it only cares about what we do. If politicians have a short-term agenda and cherry pick the data, or find a scientist who happens to agree with them, they might win in the short run, but they leave themselves vulnerable in the long run. Wise policy advisers encourage policy-makers to respect the science, and, of course to communicate evidence-based messages as effectively as possible.” (21) We wholeheartedly agree with this criticism by Fleck & Fishhoff.

It is hoped that vigilant science, media and civil society will defend the CDC's independent science-based work and urge political leaders to respect science and act accordingly, in the best interest of the people they serve. The eventual deletion of the Statement from the CDC's website is testimony to the critical need and power of such public vigilance.

## Data Availability Statement

The original contributions generated for this study are included in the article/supplementary material, further inquiries can be directed to the corresponding author/s.

## Author Contributions

All authors contributed equally to the concept, analysis and interpretations presented in the manuscript.

## Conflict of Interest

NS was employed by company The Health Bureau Ltd. The remaining authors declare that the research was conducted in the absence of any commercial or financial relationships that could be construed as a potential conflict of interest.
